# Symptomatic presentation of cervical cancer in emergency departments in California

**DOI:** 10.1007/s10552-021-01489-z

**Published:** 2021-08-23

**Authors:** Frances B. Maguire, Julianne J. P. Cooley, Cyllene R. Morris, Arti Parikh-Patel, Vanessa A. Kennedy, Theresa H. M. Keegan

**Affiliations:** 1 California Cancer Reporting and Epidemiologic Surveillance Program, University of California Davis Comprehensive Cancer Center, Sacramento, CA USA; 2 Department of Gynecology Oncology, University of California Davis Comprehensive Cancer Center, Sacramento, CA USA; 3 Center for Oncology Hematology Outcomes Research and Training (COHORT) and Division of Hematology and Oncology, University of California Davis School of Medicine, Sacramento, CA USA; 4 California Cancer Reporting and Epidemiologic Surveillance Program, University of California Davis Comprehensive Cancer Center, 1631 Alhambra Blvd., Suite 200, Sacramento, CA 95816 USA

**Keywords:** Cervical cancer, Emergency department, Symptomatic presentation, Socioeconomic status, Population-based

## Abstract

**Purpose:**

Through screening and HPV vaccination, cervical cancer can mostly be prevented or detected very early, before symptoms develop. However, cervical cancer persists, and many women are diagnosed at advanced stages. Little is known about the degree to which U.S. women may begin their diagnostic workup for cervical cancer in Emergency Departments (ED). We sought to quantify the proportion of women presenting symptomatically in the ED prior to their diagnosis with cervical cancer and to describe their characteristics and outcomes.

**Methods:**

We identified women diagnosed from 2006 to 2017 with cervical cancer in the California Cancer Registry. We linked this cohort to statewide ED discharge records to determine ED use and symptoms present at the encounter. Multivariable logistic regression models examined associations with ED use and multivariable Cox proportional hazards regression models examined associations with survival.

**Results:**

Of the more than 16,000 women with cervical cancer in the study cohort, 28% presented symptomatically in the ED prior to diagnosis. Those presenting symptomatically were more likely to have public (odds ratio [OR] 1.16; 95% confidence interval [CI] 1.06–1.27) or no insurance (OR 4.81; CI 4.06–5.71) (vs. private), low socioeconomic status (SES) (OR 1.76; CI 1.52–2.04), late-stage disease (OR 5.29; CI 4.70–5.96), and had a 37% increased risk of death (CI 1.28–1.46).

**Conclusion:**

Nearly a third of women with cervical cancer presented symptomatically, outside of a primary care setting, suggesting that many women, especially those with low SES, may not be benefiting from screening or healthcare following abnormal results.

## Introduction

Cervical cancer has both effective screening strategies (Pap and human papillomavirus [HPV] testing) [1] and since 2006 vaccines against HPV, the causative agent in the vast majority of cases [2]. After large declines in incidence and mortality, rates have plateaued since 2012 [3] with approximately 13,000 diagnoses and 4,200 deaths each year in the United States (U.S.) [4]. Many women still are diagnosed at advanced stages when survival is poor [4] despite the ability of screening to detect precancer and early-stage cancer of the cervix [1].

Not all women are benefiting from cervical cancer prevention tools. Cervical cancer screening declined from 2000 to 2015 and is below the Healthy People 2020 target [5]. HPV vaccination rates among girls by age 15 (46%) remain far behind target levels of 80% [6]. Having a primary care provider has been identified as a key strategy in cervical cancer prevention [7]. Safety-net healthcare systems face a large burden of cervical cancer patients with no prior healthcare contact, and many began their diagnostic process in the emergency department (ED), presenting with symptoms [7]. Patients with symptoms at diagnosis often have more advanced disease [8]. Nationally representative studies on cervical cancer presentation in the emergency department are lacking. Prior work has focused on ED care for patients with gynecologic cancers after their diagnosis or ED diagnostic workup among all cancer patients [9–12].

Therefore, we sought to quantify the number of women presenting symptomatically to the ED in the months prior to their cervical cancer diagnosis and describe their characteristics and survival outcomes in a large population-based cohort. Describing this population of women presenting outside of a primary care setting can highlight patient factors associated with barriers to preventive healthcare, and in turn identify opportunities for intervention.

## Methods

### Study population

We identified women diagnosed with a first primary cervical cancer from 2006 to 2017 through the California Cancer Registry (CCR) using the International Classification of Diseases (ICD) for Oncology, 3rd edition, code 27010 [13]. The CCR is a state-mandated population-based cancer surveillance system that collects incidence reports on more than 160,000 new cases of cancer diagnosed annually in California. The CCR is composed of three National Cancer Institute (NCI) Surveillance, Epidemiology, and End Results (SEER) registries that collect data on tumor characteristics, treatment, and patient demographic information. We linked the study cohort to the Office of Statewide Health Planning and Development (OSHPD) patient hospital and ED discharge records through a probabilistic linkage matching on social security number, date of birth, sex, and zip code. We included hospital inpatient discharge records in order to capture ED visits that resulted in a hospital admission.

Patients diagnosed at autopsy or death certificate only (*n* = 83) or missing date of last contact (*n* = 373) were excluded from analysis, resulting in a study population of 16,363. All analyses were overseen by the Institutional Review Board of the University of California, Davis.

### Emergency department information

OSHPD contains information on each patient admission at nonfederal acute care hospitals in California. We selected the ED visits occurring in the 6 months before cervical cancer diagnosis. The discharge records include information on principal diagnosis, principal procedure, and up to 24 secondary diagnoses and procedures based on ICD, 9th Revision (ICD-9), ICD, 10th Revision (ICD-10), and Current Procedural Terminology (CPT) codes. Diagnoses and procedures suggestive of cervical cancer were determined from OSHPD ICD-9, ICD-10, and CPT codes. The list of diagnoses and procedures were identified from consultations with physicians and final review by a gynecologist, oncologist, and co-author (Tables 4 and 5). Women were classified as having a symptom or procedure suggestive of cervical cancer in the ED within 6 months prior to diagnosis (yes/no).

### Sociodemographic and clinical characteristics

We obtained patient characteristics and treatment information from the CCR including stage and age at diagnosis, diagnosis year, health insurance type (for patients without an ED visit prior to diagnosis), race/ethnicity, neighborhood SES (nSES), rural/urban residence, comorbidity score, marital status, treatment at a NCI-designated cancer center, chemotherapy treatment, radiation treatment, and surgical treatment. From OSHPD we determined health insurance information for the patients seen in the ED prior to diagnosis.

Stage at diagnosis was assigned using the American Joint Committee on Cancer staging system rules. We categorized health insurance for patients without an ED visit from CCR information as private (HMO, PPO, fee for service, Veterans Affairs, Tricare, Medicare with supplement), public (Medicaid, Medicare with Medicaid eligibility, Medicare without supplement, county funded, Indian/public health service), uninsured, and unknown. For patients with an ED visit before diagnosis, we categorized health insurance from OSHPD information, as above, in order to best capture pre-diagnosis insurance status.

We classified race/ethnicity as non-Hispanic White (NH White), non-Hispanic Black (NH Black), Hispanic, Asian/Pacific Islander (API), American Indian, and other/unknown, based on the North American Association of Central Cancer Registries’ Hispanic and Asian/Pacific Islander Identification Algorithm (NHAPIIA) [14]. Neighborhood SES was assigned using an aggregate measure based on 2006–2010 American Community Survey data on education, occupation, unemployment, household income, poverty, rent, and home values of census tracts [15]. Rural/urban residence was based on Medical Service Study Area designations, a state-specific measure developed by the California Office of Statewide Health Planning and Development (OSHPD) to identify medically underserved areas [16].

We assessed patient comorbidities using the Charlson comorbidity index using categories of 0, 1, and 2 or more comorbidities based on sixteen medical conditions, excluding cancer diagnoses, reported in OSHPD discharge data linked to the CCR data [17]. Comorbidities could be evaluated for patients with an inpatient, emergency department, or ambulatory surgery center admission. Treatment at NCI-designated cancer centers was determined by reviewing all reporting facilities where patient treatment occurred.

### Statistical analysis

We used descriptive statistics (frequencies, percentages) to assess unadjusted associations between sociodemographic and clinical characteristics by symptomatic ED visit prior to diagnosis. Multivariable logistic regression models were used to analyze sociodemographic and clinical factors associated with pre-diagnosis symptomatic ED visits. Results are presented as adjusted odds ratios (OR) and their associated 95% confidence intervals (CI). We assessed collinearity between variables using eigenvalues and variance inflation factors. We used multivariable Cox proportional hazards regression to evaluate symptomatic presentation in the ED prior to diagnosis and overall (OS) and cancer-specific survival (CSS), adjusting for stage, age, diagnosis year, health insurance, race/ethnicity, nSES, rural/urban residence, comorbidity score, marital status, treatment at NCI-designated cancer center, and receipt of chemotherapy, radiation, and surgery. Survival time was calculated as days from the date of diagnosis to the date of death from any cause for OS and to the date of death from cancer for CSS or the date of last follow-up. We assessed proportional hazards assumptions with tests based on Schoenfeld residuals and inspection of the survival curves [survival function vs survival time and log (− log) of the survival function versus the log of time] for all variables in the model. Variables that violated this assumption (chemotherapy, radiation) were included as stratifying variables. Results are presented as adjusted hazard ratios (HR) and their associated 95% CIs. Analyses were conducted using SAS software version 9.4 (SAS Institute Inc., Cary, North Carolina).

## Results

Of the 16,363 patients in the study cohort, 5,545 (33.9%) had an ED visit in the 6 months before diagnosis. Of those who visited the ED, 4,590 (28.1% of the study cohort) had a symptom or procedure suggestive of cervical cancer and 44.8% of these symptomatic women had more than one ED visit in the 6 months before their diagnosis. Compared to those without symptomatic ED visits, women with symptomatic visits had more late-stage disease (stages III/IV), were older (> 50 years), had public or no insurance, were NH Black, unmarried, and resided in the lowest SES neighborhoods (Table 1).

**Table 1:** Characteristics of cervical cancer patients by emergency department (ED) visit status before diagnosis (0–6 months), 2006–2017, California, *n* = 16,363

Characteristics	Had symptomatic^a^ ED visit % (*n*)	No symptomatic ED visit % (*n*)	Unknown ED visit status % (*n*)
	28.1%, *n* = 4,590	57.3%, *n* = 9,377	14.6%, *n* = 2,396
Stage			
I	25.2% (1156)	54.7% (5129)	47.3% (1134)
II	12.7% (581)	11.5% (1077)	10.9% (261)
III	28.6% (1312)	17.3% (1620)	17.9% (429)
IV	26.1% (1199)	9.8% (918)	11.7% (281)
Unknown	7.5% (342)	6.8% (633)	12.1% (291)
Age group, years			
≤ 39	21.4% (980)	29.3% (2743)	30.6% (732)
40–50	27.7% (1271)	27.8% (2607)	32.2% (771)
51–60	23.1% (1062)	19.9% (1867)	19.2% (461)
61–75	18.7% (860)	17.3% (1620)	12.8% (307)
> 75	9.1% (417)	5.8% (540)	5.2% (125)
Year of diagnosis			
2006–2010	43.8% (2010)	43.6% (4084)	34.4% (824)
2011–2013	24.7% (1132)	24.6% (2311)	23.1% (554)
2014–2017	31.5% (1448)	31.8% (2982)	42.5% (1018)
Health insurance			
Private	48.1% (2207)	61.1% (5725)	25.6% (613)
Public	37.2% (1709)	33.1% (3105)	58.1% (1393)
Uninsured	13.5% (618)	2.8% (263)	7.3% (174)
Unknown	1.2% (56)	3.0% (284)	9.0% (216)
Race/ethnicity			
NH White	39.9% (1833)	43.9% (4120)	14.7% (353)
NH Black	8.6% (394)	5.6% (527)	3.6% (86)
Hispanic	36.2% (1661)	32.3% (3031)	66.4% (1592)
Asian/Pacific Islander	13.8% (632)	16.3% (1528)	12.1% (291)
American Indian	1.2% (53)	0.8% (78)	0.3% (6)
Other/Unknown	0.4% (17)	1.0% (93)	2.8% (68)
Neighborhood SES quintile			
1 Lowest	30.8% (1415)	20.8% (1952)	36.9% (885)
2	24.3% (1114)	21.6% (2025)	25.8% (618)
3	19.3% (885)	21.5% (2016)	16.8% (403)
4	15.8% (723)	19.7% (1851)	12.0% (288)
5 Highest	9.9% (453)	16.3% (1533)	8.4% (202)
Rural/Urban location			
Rural	14.0% (641)	13.1% (1227)	8.4% (202)
Urban	86.0% (3949)	86.9% (8150)	91.6% (2194)
Charlson comorbidity score			
0	61.7% (2833)	67.3% (6308)	0% (0)
1	19.0% (874)	13.7% (1283)	0% (0)
> 1	19.0% (873)	5.7% (531)	0% (0)
Unknown	0.2% (10)	13.4% (1255)	100.0% (2396)
Marital status			
Married	36.6% (1679)	46.9% (4396)	36.1% (866)
Not married	60.4% (2771)	48.3% (4531)	52.0% (1247)
Unknown	3.1% (140)	4.8% (450)	11.8% (283)

*NH* non-Hispanic, *SES* socioeconomic status

^a^For a full list of symptoms and procedures see Tables 4 and 5

Multivariable logistic regression analysis demonstrated that increasing stage (vs. stage I) (stage II: OR 2.26; CI 1.98–2.58; stage III: OR 3.26; CI 2.94–3.62; stage IV: OR 5.29; CI 4.70–5.96), older age (vs. < 40 years) (40–50 years: OR 1.21; CI 1.08–1.35; > 75 years: OR 1.38; CI 1.16–1.64), public insurance (OR 1.16; CI 1.06–1.27) and no insurance (OR 4.81; CI 4.06–5.71) (vs. private insurance), lower nSES (vs. highest nSES quintile) (lowest quintile: OR 1.76; CI 1.52–2.04; lower middle quintile: OR 1.47; CI 1.27–1.70; middle quintile: OR 1.21; CI 1.04–1.40), more comorbidities (vs Charlson score of 0) (Charlson score 1: OR 1.28; CI 1.15–1.42; Charlson score > 1: OR 2.58; CI 2.27–2.94), and being unmarried (vs. married) (OR 1.25; CI 1.15–1.36) were associated with increased odds of symptomatic presentation in the ED prior to diagnosis. Diagnosis in more recent years, 2011–2017 (vs. 2006–2010) was associated with decreased odds of symptomatic presentation in the ED (OR 0.88–0.89; CI 0.80–0.99) (Table 2).

**Table 2:** Multivariable-adjusted^a^ odds ratios (OR) and 95% confidence intervals (CI) of associations with emergency department (ED) care for symptoms of cervical cancer prior to diagnosis^b^, 2006–2017, California

Characteristics	ED visit with cervical cancer symptoms prior to diagnosis (vs no ED visit)	
	OR (95% CI)	*p* value
Stage		
I	Reference	
II	2.26 (1.98, 2.58)	** < 0.001**
III	3.26 (2.94, 3.62)	** < 0.001**
IV	5.29 (4.70, 5.96)	** < 0.001**
Unknown	3.01 (2.54, 3.56)	** < 0.001**
Age group, years		
≤ 39	Reference	
40–50	1.21 (1.08, 1.35)	**0.001**
51–60	1.06 (0.93, 1.19)	0.382
61–75	0.98 (0.86, 1.12)	0.760
> 75	1.38 (1.16, 1.64)	** < 0.001**
Year of diagnosis		
2006–2010	Reference	
2011–2013	0.89 (0.80, 0.99)	**0.025**
2014–2017	0.88 (0.80, 0.96)	**0.006**
Health insurance		
Private	Reference	
Public	1.16 (1.06, 1.27)	**0.001**
Uninsured	4.81 (4.06, 5.71)	** < 0.001**
Unknown	0.56 (0.40, 0.77)	** < 0.001**
Race/ethnicity		
NH White	Reference	
NH Black	1.18 (1.00, 1.39)	0.050
Hispanic	1.1 (0.99, 1.21)	0.063
Asian/Pacific Islander	0.93 (0.83, 1.06)	0.284
American Indian	1.36 (0.90, 2.04)	0.142
Other/Unknown	0.92 (0.51, 1.65)	0.787
Neighborhood SES quintile		
1 Lowest	1.76 (1.52, 2.04)	** < 0.001**
2	1.47 (1.27, 1.70)	** < 0.001**
3	1.21 (1.04, 1.40)	**0.013**
4	1.16 (1.00, 1.35)	0.051
5 Highest	Reference	
Rural/Urban location		
Urban	Reference	
Rural	1.02 (0.90, 1.14)	0.784
Charlson comorbidity score		
0	Reference	
1	1.28 (1.15, 1.42)	** < 0.001**
> 1	2.58 (2.27, 2.94)	** < 0.001**
Unknown	0.01 (0.01, 0.02)	** < 0.001**
Marital status		
Married	Reference	
Not married	1.25 (1.15, 1.36)	** < 0.001**
Unknown	0.92 (0.73, 1.15)	0.463

Boldface indicates statistical significance (*p* < 0.05)

^a^Adjusted for all variables in the table

^b^In the 6 months before diagnosis; For a full list of symptoms/procedures see Tables 4 and 5

*NH* non-Hispanic, *SES* socioeconomic status

Table 3 shows results of multivariable Cox proportional hazards analyses. Women presenting symptomatically in the ED in the 6 months prior to diagnosis (vs. those without symptomatic ED visits) had a 37% increased risk of death (HR 1.37; CI 1.28–1.47). Other factors associated with worse OS and CSS included increasing stage (*p*-values < 0.001), older age groups (> 60 years) (*p*-values ≤ 0.008), more comorbidity (*p*-values < 0.001), being unmarried (*p*-values ≤ 0.015), and treatment at non-NCI -designated cancer centers (*p*-values < 0.001). Being Hispanic or API (vs, NH White) was associated with a lower risk of death (*p*-values < 0.001).

**Table 3:** Multivariable-adjusted^a^ hazard ratios (HR) and 95% confidence intervals (CI) for associations between emergency department (ED) care for symptoms of cervical cancer prior to diagnosis^b^ and mortality, 2006–2017, California

Characteristics	Overall mortality		Cancer-specific mortality	
	HR (95% CI)	*p* value	HR (95% CI)	*p* value
Symptoms at the ED				
No	Reference			
Yes	1.37 (1.28, 1.46)	** < 0.001**	1.37 (1.28, 1.47)	** < 0.001**
Unknown	0.86 (0.74, 0.99)	**0.034**	0.78 (0.67, 0.92)	**0.003**
Stage				
I	Reference			
II	1.98 (1.76, 2.24)	** < 0.001**	2.49 (2.15, 2.87)	** < 0.001**
III	3.51 (3.16, 3.90)	** < 0.001**	4.73 (4.17, 5.35)	** < 0.001**
IV	8.13 (7.32, 9.03)	** < 0.001**	11.71 (10.34, 13.26)	** < 0.001**
Unknown	3.04 (2.69, 3.44)	** < 0.001**	4.10 (3.55, 4.73)	** < 0.001**
Age group, years				
≤ 39	Reference			
40–50	1.12 (1.02, 1.22)	**0.015**	1.05 (0.95, 1.15)	0.342
51–60	1.23 (1.12, 1.34)	** < 0.001**	1.06 (0.96, 1.16)	0.281
61–75	1.51 (1.38, 1.66)	** < 0.001**	1.15 (1.04, 1.27)	**0.008**
> 75	2.64 (2.36, 2.94)	** < 0.001**	1.87 (1.65, 2.12)	** < 0.001**
Year of diagnosis				
2006–2010	Reference			
2011–2013	1.05 (0.98, 1.12)	0.156	1.05 (0.97, 1.13)	0.212
2014–2017	0.95 (0.89, 1.02)	0.165	0.94 (0.87, 1.01)	0.086
Health insurance				
Private	Reference			
Public	1.03 (0.97, 1.10)	0.287	0.98 (0.92, 1.05)	0.606
Uninsured	0.99 (0.89, 1.10)	0.888	0.96 (0.86, 1.08)	0.538
Unknown	1.19 (1.01, 1.41)	**0.041**	1.15 (0.95, 1.39)	0.147
Race/ethnicity				
NH White	Reference			
NH Black	1.08 (0.97, 1.20)	0.145	1.07 (0.95, 1.20)	0.250
Hispanic	0.85 (0.79, 0.91)	** < 0.001**	0.86 (0.80, 0.93)	** < 0.001**
Asian/Pacific Islander	0.81 (0.75, 0.88)	** < 0.001**	0.84 (0.77, 0.93)	** < 0.001**
American Indian	0.86 (0.63, 1.16)	0.311	0.85 (0.61, 1.19)	0.347
Other/Unknown	0.13 (0.06, 0.29)	** < 0.001**	0.06 (0.01, 0.23)	** < 0.001**
Neighborhood SES quintile				
1 Lowest	1.13 (1.02, 1.25)	**0.018**	1.09 (0.98, 1.22)	0.120
2	1.08 (0.97, 1.19)	0.153	1.02 (0.91, 1.15)	0.683
3	1.04 (0.93, 1.15)	0.509	1.02 (0.91, 1.14)	0.723
4	0.95 (0.86, 1.06)	0.397	0.95 (0.85, 1.07)	0.430
5 Highest	Reference			
Rural/Urban location				
Rural	1.02 (0.94, 1.11)	0.660	1.01 (0.92, 1.11)	0.839
Urban	Reference			
Charlson comorbidity score				
0	Reference			
1	1.30 (1.21, 1.41)	** < 0.001**	1.21 (1.11, 1.32)	** < 0.001**
> 1	1.91 (1.77, 2.07)	** < 0.001**	1.67 (1.53, 1.83)	** < 0.001**
Unknown	1.14 (1.02, 1.28)	**0.022**	1.11 (0.98, 1.25)	0.110
Marital status				
Married	Reference			
Not married	1.08 (1.02, 1.15)	**0.008**	1.09 (1.02, 1.16)	**0.015**
Unknown	0.93 (0.80, 1.08)	0.335	0.92 (0.78, 1.08)	0.317
Treatment at NCI-designated Cancer Center				
Yes	Reference			
No	1.28 (1.20, 1.37)	** < 0.001**	1.31 (1.21, 1.41)	** < 0.001**

Boldface indicates statistical significance (*p* < 0.05)

^a^Adjusted for all variables in the table and surgery treatment; Chemotherapy and radiation treatment were included as strata because of non-proportional hazards

^b^In the 6 months before diagnosis; For a full list of symptoms/ procedures see Tables 4 and 5

*NCI* National Cancer Institute, *NH* non-Hispanic, *SES* socioeconomic status

**Table 4:** Diagnoses^a^ suggestive of cervical cancer recorded in the emergency department (ED) or subsequent hospital stay in the 6 months prior to diagnosis with cervical cancer among the 5,545 patients with an ED visit

Diagnoses	% (*n*)
Disorders of the genital tract	49.1% (2724)
Dysplasia of cervix, erosion of cervix	
Polyp of cervix	
Inflammatory disease of cervix, vagina, or vulva	
Noninflammatory disorders of cervix, vagina, vulva, or perineum	
Leukorrhea, noninfective	
Dyspareunia, post coital bleeding	
Excessive or frequent menstruation, dysmenorrhea	
Metrorrhagia, postmenopausal bleeding	
Genital fistula	
Hypertrophy of uterus	
Disorders of the urinary tract	18.5% (1026)
Hydronephrosis	
Hydroureter, ureter obstruction	
Urinary obstruction	
Bladder obstruction	
Retention of urine	
Urinary hesitancy, frequency, dysuria	
Oliguria and anuria	
Hematuria	
Renal colic	
Kidney failure	
Genitourinary neoplasm	42.7% (2367)
Carcinoma in situ cervix	
Malignant neoplasm of cervix	
Malignant neoplasm, pelvis	
Malignant neoplasm of endocervix, exocervix	
Malignant or benign neoplasm vagina, lower uterine segment, female genitalia	
Malignant or benign neoplasm bladder, ureter, urethra	
Pain or mass	21.0% (1163)
Abdominal pain	
Abdominal mass	
Pelvic pain	
Pelvic mass	
Low back pain	
Neoplasm-related pain	
Ascites	
Blood loss/anemia	36.1% (2001)
Acute post-hemorrhagic anemia	
Iron deficiency anemia secondary to blood loss	
Anemia in neoplastic disease	
Anemia of other chronic disease	
Other iron deficiency anemias	

^a^Based on ICD9 and ICD10 diagnosis codes

**Table 5:** Procedures^a^ suggestive of cervical cancer recorded in the emergency department or subsequent hospital stay in the 6 months prior to diagnosis with cervical cancer among the 5,545 patients with an ED visit

Procedures	% (*n*)
Operations on the genital tract	34.3% (1904)
Operations on cervix (excision, resection, drainage)	
Excision and incision of uterus including hysterectomy	
Diagnostic procedures (biopsy, excision) on vulva, vagina, and cul-de-sac	
Hemostatic agent for nonobstetrical vaginal hemorrhage	
Endometrial sampling, extraction	
Operations on urinary system	14.3% (794)
Nephrostomy, replacement of nephrostomy tube	
Operations (excision, drainage, dilation,) on ureter (including stent placement), urinary bladder, urethra, kidney	
Ureteral or bladder catheterization	
Diagnostic procedures (cystourethroscopy, ureteroscopy, pyeloscopy) of kidney, bladder, ureter, urethra	
Operations on lymph nodes	6.8% (376)
Biopsy, excision, or dissection of lymph node (inguinal, pelvis)	
Diagnostic and therapeutic procedures	39.2% (2171)
Gynecological examination: pelvic exam	
Diagnostic proctoscopy, sigmoidoscopy, colonoscopy	
Transfusion of blood	
Injection or infusion of cancer chemotherapeutic substance	
Imaging urinary system (X-ray, ultrasound, other)	
Imaging female reproductive system (X-ray, ultrasound, other)	
Imaging abdomen, pelvis (CT, MRI, ultrasound, other)	

^a^Based on ICD9, ICD10, and CPT4 procedure codes

## Discussion

In our population-based study of over 16,000 women with cervical cancer, the 28% of women presenting symptomatically in the ED within 6 months prior to diagnosis were three–five times more likely to be diagnosed with stage III or IV disease and had worse overall and cervical cancer-specific survival. Women presenting symptomatically in the ED were nearly five times more likely to have no insurance and nearly two times more likely to reside in low-SES neighborhoods. This is consistent with prior studies showing poor primary care access and use of the ED for usual source of care among low-SES populations [7, 18]. Nearly half of women presenting symptomatically had more than one ED visit. Those diagnosed in more recent years were less likely to present symptomatically in the ED, which may relate to changes in healthcare policies in California to improve access to care. Overall, our study indicates that many women, especially low-SES women, are likely not benefiting from cervical cancer screening or care following an abnormal screen, have poor access to healthcare, and are diagnosed with advanced-stage disease outside of a primary care setting.

The 28% of our study cohort presenting symptomatically is on the high end of ranges previously described. Other U.S. studies, not focused on cervical cancer, have found the proportion of cancer patients beginning their diagnostic workup in the ED ranging from 5% in 1992 to 32% in 2016 [11, 12, 19]. International studies on all cancers have reported diagnoses in the ED ranging from 14% of cancer patients in 2013 to 20% in 2016 [20, 21]. Cancer presentation in the ED has been associated with less favorable patient characteristics such as advanced-stage disease and comorbid conditions, consistent with our findings [7, 12, 18].

Early cancer diagnosis improves survival [22]. Women diagnosed with cervical cancer as a result of screening are more likely to be asymptomatic and have early-stage disease [8]. Screening rates vary by socioeconomic status with lower screening rates among poor and less educated women [5, 23]. Screening utilization is approximately 20% lower in women with less than a high school education compared to college graduates, 15% lower in women earning less than 139% of federal poverty level compared to those earning greater than 400%, and 20% lower in women without a usual source of healthcare [5]. This may explain our findings of increased odds of symptomatic presentation in the ED among women residing in low-SES neighborhoods and among those with public or no insurance. In addition, unmarried women were more likely to present symptomatically, consistent with findings showing delayed cervical cancer diagnosis among unmarried women [24]. By age group, women > 75 had the greatest likelihood of symptomatic presentation in the ED. High cervical cancer incidence and mortality in older women has been noted, but current guidelines do not recommend cervical cancer screening in those over age 65 who have had adequate negative prior screening within the past 10 years, and no history of CIN2+ (cervical intraepithelial neoplasia grade 2 or more) within the past 25 years [25–27]. Our study was unable to determine whether women in this age category met the criteria to exit screening.

We found that women diagnosed in more recent years had a decreased likelihood of symptomatic presentation in the ED. From 2010 to 2014, several policies were enacted in California that may have improved access to preventive health care in our study cohort. Starting in late 2010, the Affordable Care Act (ACA) Dependent Care Expansion permitted young adults to remain on their parents’ health insurance until age 26 [28]. From 2011 to 2013, the Low-Income Health Plan extended Medicaid coverage to uninsured adults ages 19–64 with a family income up to 200% of the Federal Poverty Level [29]. In January 2014, the full ACA was implemented, expanding Medicaid coverage and providing a private health insurance marketplace with subsidies for eligible individuals [30]. Expanded healthcare provisions may have resulted in more primary care screening with follow-up and less reliance on the ED during the later years of our study period.

We did not observe differences by race/ethnicity for symptomatic presentation in the ED, unlike previous studies such as Livingwood et al. that found higher proportions of Black patients beginning their cancer diagnoses in the ED [12]. However, we did find better survival among Hispanic and API women compared to NH White women. Our survival findings are consistent with prior studies including those from Nghiem et al. that found that Asian American women with cervical cancer (vs. NH White women) had higher survival rates, and Patel et al. that found that Hispanic women had a survival advantage compared to non-Hispanic White women with cervical cancer [31, 32]. Factors found to be associated with better survival in Asian and/or Hispanic women include higher SES, having family support (spouses, domestic partners), and better adherence to recommended treatment [24, 32, 33]. The effect of nativity could also play a role. Studies have revealed that foreign-born Hispanic women have better survival than their U.S. born counterparts (Hispanic paradox) [34, 35]. It has been speculated that Asian American women may be subject to a similar effect [32]. Further study is needed to better understand these associations.

Women in our study presenting symptomatically in the ED had a 37% increased risk of death after controlling for factors known to be associated with survival, including stage at diagnosis, age, comorbidity, and treatment at NCI-designated cancer centers [22, 36, 37]. This is in agreement with other studies that have found later stage disease and worse survival among cancer patients beginning their diagnostic workup in the ED [12]. However, prior studies have not evaluated cervical cancer.

Our study had some limitations. We lacked information on cervical cancer screening and HPV vaccination uptake among our cohort. Although it is likely that a large percentage of our cohort presenting symptomatically with advanced-stage disease had not been screened, we were unable to confirm this. Additionally, we were unable to identify patients presenting symptomatically in outpatient settings. Finally, 14.6% of our study cohort had unknown ED visit status. These patients had missing social security numbers and could not be linked to ED records. It is therefore possible that we underestimated the number of women with symptomatic ED visits prior to diagnosis. Although the women with unknown ED visit status had some similarities to those with symptomatic visits such as more public and no insurance and high percentages residing in low-SES neighborhoods, they had similar proportions of late-stage disease to those without symptomatic ED visits and they had better OS and CSS. In sensitivity analyses combining the unknowns with those without ED visits, OS and CSS remained unchanged. Despite these limitations, our study was able to estimate the proportion of women with cervical cancer who presented symptomatically prior to their diagnosis and to describe their characteristics and outcomes in a large ethnically and geographically diverse state.

Nearly one-third of cervical cancer patients in California present symptomatically over 50 years after the introduction of Pap tests and over 15 years after the creation of the National Breast and Cervical Cancer Early Detection Program [38]. Additionally, some patients in our cohort were eligible for HPV vaccination. Early diagnosis through screening, before symptoms appear, can significantly decrease morbidity and mortality in this highly preventable disease [1]. Symptomatic ED use, more likely in women residing in low-SES neighborhoods or with public or no insurance, suggests poor access to preventive healthcare. Almost half of the women presenting symptomatically in our study had more than one ED visit prior to their diagnosis. ED use patterns can reflect the healthcare delivery of a community and be a marker of deficiencies in the primary care infrastructure [11]. Our findings suggest that efforts should be made to increase adherence to cervical cancer screening guidelines, increase uptake of HPV vaccination, and ensure proper follow-up care especially among low-SES women. Further studies investigating the barriers to care low-SES women face and strategies to expand preventive care are warranted.

## Data Availability

Per contract with the State of California, the data used in this study cannot be shared. However, investigators can apply directly to the California Cancer Registry to request these data (https://www.ccrcal.org/retrieve-data/).

## Code availability

Programming codes used for the analysis can be requested from the corresponding author.
